# The M2 Protein of the Influenza A Virus Interacts with PEX19 to Facilitate Virus Replication by Disrupting the Function of Peroxisome

**DOI:** 10.3390/v16081309

**Published:** 2024-08-16

**Authors:** Tanbin Liu, Libin Liang, Pu Zhao, Weipeng Lin, Yichao Zhuang, Li Jiang, Hualan Chen, Chengjun Li

**Affiliations:** 1 College of Veterinary Medicine, China Agricultural University, Beijing 100193, China; tanbinliucaas@gmail.com (T.L.); b20223050422@cau.edu.cn (P.Z.); 2 State Key Laboratory for Animal Disease Control and Prevention, Harbin Veterinary Research Institute, Chinese Academy of Agricultural Sciences, Harbin 150069, China; linweipeng1995@163.com (W.L.); zhuangachao@yeah.net (Y.Z.); jiangli@caas.cn (L.J.); 3 College of Veterinary Medicine, Shanxi Agricultural University, Jinzhong 030801, China; lianglibin@sxau.edu.cn

**Keywords:** PEX19, influenza A virus, M2, peroxisome, virus replication, type III interferon

## Abstract

The peroxisomal biogenesis factor 19 (PEX19) is necessary for early peroxisomal biogenesis. PEX19 has been implicated in the replication of a variety of viruses, but the details pertaining to the mechanisms of how PEX19 engages in the life cycle of these viruses still need to be elucidated. Here, we demonstrated that the C terminus of PEX19 interacted with the cytoplasmic tail region of the M2 protein of the influenza A virus (IAV) and inhibited the viral growth titers. IAV infection or PEX19 knockdown triggered a reduction in the peroxisome pool and led to the accumulation of ROS and cell damage, thereby creating favorable conditions for IAV replication. Moreover, a reduction in the peroxisome pool led to the attenuation of early antiviral response mediated by peroxisome MAVS and downstream type III interferons. This study also showed that the interaction between IAV M2 and PEX19 affected the binding of PEX19 to the peroxisome-associated protein PEX14 and peroxisome membrane protein 24 (PMP24). Collectively, our data demonstrate that host factor PEX19 suppresses the replication of the IAV, and the IAV employs its M2 protein to mitigate the restricting role of PEX19.

## 1. Introduction

The influenza A virus (IAV) can infect a variety of species of birds and mammals [1]. It is the causative agent of seasonal epidemics and occasional pandemics in humans. In addition to the contemporary H1N1 and H3N2 seasonal influenza viruses, the threats posed by the spillover of various subtypes of avian influenza viruses (e.g., H5N1, H5N6, H7N9, H9N2, H3N8, and H10N8) to humans are not neglectable [2,3,4,5,6,7]. The matrix protein 2 (M2) of the IAV is a type III membrane protein that exists as a homotetramer on the envelope of virions. M2 has no signal peptide and is composed of three distinct domains: ectodomain (ED, amino acids 1–24), transmembrane domain (TM, amino acids 25–43), and cytoplasmic tail domain (CT, amino acids 44–97) [8,9]. M2 is involved in several stages of the IAV replication cycle. During virus entry, the proton channel activity of M2 leads to the dissociation of the viral ribonucleoprotein (vRNP) complexes from M1 and the lipid bilayers, thus completing the uncoating process [10]. The proton channel activity of M2 also functions to raise the pH of the trans-Golgi network (TGN), thereby preventing the premature low-pH conformational change of viral hemagglutinin (HA) to ensure proper viral assembly and release [11,12]. In the late stages of viral replication, M2 is required to maintain the stability of the budzone and assist in the final release of viral particles [13,14].

M2 interacts with multiple host cellular factors while executing its function in the viral life cycle. It interacts with LC3 and inhibits the fusion of autophagosomes with lysosomes, thereby enhancing the stability of the progeny virus [15]. M2 binds to the autophagy-related factor Atg6/Beclin-1 via amino acids 1–60, which also results in the blockage of fusion between autophagosomes and lysosomes [16]. Annexin A6 (ANXA6) interacts with M2, which impairs the budding and release of progeny viruses [17]. The host factor Na^(+)^/K^(+)^-ATPase β1 subunit (ATP1B1) binds to the cytoplasmic tail domain of M2, which promotes the replication of the IAV [18]. The ubiquitin protein ligase E3 component N-Recognin 4 (UBR4) interacts with the transmembrane domain and cytoplasmic tail of M2, which facilitates the cell surface translocation of viral proteins, i.e., HA, NA, and M2 proteins, in the late stage of IAV infection [19]. The N-terminal internal-deletion isoform of the 6A subunit of the transport protein complex (TRAPPC6A) interacts with the highly conserved 96L residue of M2, modulating M2 trafficking to the cell membrane and promoting viral replication and pathogenicity [20].

PEX19, also known as PXF, is a hydrophilic farnesylated protein comprising 299 amino acids [21]. PEX19 is bimodally distributed between the cytoplasm and the peroxisome at a steady state, with the cytoplasm containing the majority of the protein [22]. Farnesylated PEX19 is partially anchored in the peroxisomal membrane, exposing its N-terminal part to the cytosol [23]. PEX19 is required for early peroxisome biogenesis and functions as a receptor and chaperone of peroxisomal membrane proteins (PMPs) [24]. It recognizes PMPs in the cytoplasm via a globular domain at the C terminus and delivers them to the peroxisome membrane by binding the PEX3 receptor [25].

PEX19 is involved in the life cycle of distinct viruses. PEX19 is significantly upregulated in EBV-infected cells [26]. vMIA, a human cytomegalovirus-encoded protein, interacts with PEX19, which may contribute to evading the peroxisomal MAVS-dependent antiviral response [27]. The capsid proteins of flaviviruses, e.g., West Nile and dengue viruses, interact with PEX19, leading to impaired peroxisome biogenesis and early antiviral signaling [28]. The viral FLICE-inhibitory protein (vFLIP) of human herpesvirus 8 (HHV-8) is targeted to peroxisomes in a PEX19-dependent manner to establish or maintain latency [29]. However, whether PEX19 plays a role in the replication of the IAV is still unknown.

In this study, we used the yeast two-hybrid (Y2H) system to screen for host proteins that interact with the IAV M2 protein and identified PEX19 as a potential binding partner. The M2-PEX19 interaction was validated by co-immunoprecipitation (co-IP) and confocal microscopy. PEX19 appeared to reduce the replication of the IAV in vitro. IAV infection or PEX19 knockdown triggered a reduction in the peroxisome pool and led to the accumulation of ROS and cell damage, which created favorable conditions for IAV replication. Moreover, a reduction in the peroxisome pool led to the attenuation of an early antiviral response mediated by peroxisome MAVS and downstream type III interferons. A mechanism study revealed that the interaction between IAV M2 and PEX19 impaired the binding of PEX19 to the peroxisome-associated proteins.

## 2. Materials and Methods

### 2.1. Cells and Viruses

Human embryonic kidney cells (HEK293T, ATCC CRL-3216), human lung carcinoma cells (A549, ATCC CCL-185), and Madin–Darby canine kidney (MDCK, ATCC CCL-34) cells were cultured as described previously [30].

A/WSN/1933 (WSN, H1N1), A/Anhui/2/2005 (AH05, H5N1) and A/Anhui/1/2013 (AH13, H7N9) were propagated on MDCK cells or 10-day-old embryonated chicken eggs as described previously [31].

All experiments with AH05 (H5N1) and AH13 (H7N9) viruses were conducted within the ABSL3+ facility in the Harbin Veterinary Research Institute (HVRI) of the Chinese Academy of Agricultural Sciences (CAAS), which is approved for such use by the Ministry of Agriculture and Rural Affairs of the People’s Republic of China and the China National Accreditation Service for Conformity Assessment.

### 2.2. Yeast Two-Hybrid Assay

The matchmaker yeast two-hybrid system (Clontech, Mountain View, CA, USA) was used to screen for potential interacting partners of M2 of the A/Sichuan/1/2009 (SC09, H1N1) virus, as described previously [20], among which PEX19 was identified as a potential binding partner of M2. To confirm the interaction between M2 and PEX19, the bait plasmid pGBKT7-SC09M2 and prey plasmid pGADT7-PEX19 were co-transformed into the Y2HGold yeast strain. The co-transformation of pGBKT7-p53 (BD-p53) and pGADT7-T served as a positive control. The co-transformation of pGBKT7-Lamin and pGADT7-T served as a negative control.

### 2.3. Plasmids

The PEX19 gene was amplified from total cellular mRNAs of A549 cells by reverse transcription PCR (RT-PCR) with Superscript III reverse transcriptase (Invitrogen, Carlsbad, CA, USA). The open reading frame (ORF) of PEX19 was subsequently cloned into the pCAGGS expression vector. Plasmid pCAGGS-Myc-PEX19 was generated by inserting the PEX19 ORF fused with a Myc tag sequence in the N terminus into the pCAGGS vector. The truncation constructs, i.e., pCAGGS-Myc-PEX19 (1–87) and pCAGGS-Myc-PEX19 (88–299), were created in the same way. Plasmid pCAGGS-SC09M2 was constructed as described previously [20]. The full-length or truncated ORF of SC09 (H1N1) M2 was cloned into pCAGGS with a GST or Flag tag at the N terminus. The BM2 sequence of the B/Jilin/20/2003 virus (GenBank accession number CY033829.1) was synthesized (Comate Bioscience, Changchun, China) and inserted into the pEGFP-C1 vector to generate the construct pEGFP-C1-BM2.

### 2.4. Antibodies

The primary antibodies used in this study include rabbit anti-Flag polyclonal antibody (pAb) (F7425; Sigma-Aldrich, Saint Louis, MO, USA), mouse anti-Flag monoclonal antibody (mAb) (F3165; Sigma-Aldrich), rabbit anti-Myc pAb (C3965; Sigma-Aldrich), mouse anti-Myc mAb (M4439; Sigma-Aldrich), mouse anti-actin mAb (sc-47778; Santa Cruz, Dallas, TX, USA), rabbit anti-GAPDH pAb (10494-1-AP; Proteintech, Wuhan, China), rabbit anti-GFP pAb (AG279; Beyotime Biotech, Shanghai, China), mouse anti-GFP mAb (ab1218; Abcam, Cambridge, MA, USA), rabbit anti-GST pAb (A00097; Genscript, Nanjing, China), mouse anti-GST mAb (A00865; Genscript), rabbit anti-V5 pAb (14440-1-AP; Proteintech), mouse anti-V5 mAb (A01724; Genscript), rabbit anti-M2 pAb (GTX125951; GeneTex, Irvine, CA, USA), mouse anti-M2 mAb (ab5416; Abcam), rabbit anti-PEX19 pAb (GTX110721; GeneTex), mouse anti-PEX19 mAb (GT554; GeneTex), and mouse anti-PMP70 mAb (ab211533; Abcam). DyLight 680 goat anti-mouse IgG (H+L) (RS23710; Immunoway, Plano, TX, USA) and DyLight 800 goat anti-rabbit IgG (H+L) (RS23920; Immunoway) were used for Western blotting. Alexa Fluor 488 goat anti-mouse IgG (H+L) (A-11029; Invitrogen) and Alexa Fluor 633 goat anti-rabbit IgG (H+L) (A-21071; Invitrogen) were used for confocal microscopy.

### 2.5. Co-Immunoprecipitation Assay

HEK293T cells cultured in 6-well plates were transfected with the combination of plasmids, as indicated, using the Lipofectamine LTX with Plus reagents (Invitrogen). Forty-eight hours after transfection, the cells were washed three times with cold phosphate-buffered saline (PBS) (pH 7.4) and lysed in IP buffer [25 mM Tris-HCl (pH 7.4), 150 mM sodium chloride, 1% NP-40, 1 mM EDTA, and 5% glycerol] (Pierce, Rockford, IL, USA) containing a complete protease inhibitor (Roche Diagnostics, Mannheim, Germany) on ice for 30 min. The cell lysates were centrifuged for 10 min at 12,000 rpm, and the supernatant was incubated with the appropriately diluted primary antibodies and protein G-agarose beads (Roche) at 4 °C for 6–8 h. The beads were washed four times with 1 mL of wash buffer [25 mM Tris-HCl (pH 7.4), 150 mM sodium chloride, and 1% NP-40]. The bound proteins were resolved by SDS-PAGE and detected by Western blotting.

### 2.6. Western Blotting

Protein samples were separated by SDS-PAGE and electrotransferred onto nitrocellulose membranes (GE Healthcare, Pittsburgh, PA, USA). Blots were blocked for 1 h at room temperature (RT) with 5% skim milk in PBS containing 0.05% Tween 20 (PBST), followed by incubation overnight at 4 °C with the indicated primary antibodies diluted in PBST containing 2% bovine serum albumin. After washing four times with PBST, the blots were incubated with DyLight 680 goat anti-mouse IgG (H+L) (1:10,000) or DyLight 800 goat anti-rabbit IgG (H+L) (1:10,000) at 4 °C for 1 h and scanned using an Odyssey CLX infrared imaging system (Li-Cor BioScience, Lincoln, NE, USA).

### 2.7. Cell Viability Assay

Cell viability was determined by measuring intracellular ATP levels using a CellTiter-Glo kit (Promega, Madison, WI, USA) according to the manufacturer’s instructions. Briefly, A549 cells were seeded into opaque-walled 96-well plates and transfected with scrambled siRNA or siRNA targeting PEX19 at a concentration of 40 nM. Then, 36 h after transfection, cell lysis was induced by adding 100 µL of CellTiter-Glo reagent directly into each well and rocking at RT for 10 min on a shaker. The luminescence was then detected using a GloMax 96 microplate luminometer (Promega).

### 2.8. siRNA Knockdown and Virus Infection

Scrambled siRNA or specific siRNA targeting PEX19 (PEX19 siRNA1, sense: 5′-GCAGCAGCUACAAGAUUUATT-3′, antisense: 5′-UAAAUCUUGUAGCUGCUGCTT-3′; PEX19 siRNA2, sense: 5′-GGAACUAUUCGACAGUGAATT, antisense: 5′-UUCACUGUCGAAUAGUUCCTT) was transfected into A549 cells, and the knockdown efficiency of PEX19 in the cell lysates was determined by Western blotting 48 h after transfection. In a separate experiment, A549 cells were transfected with scrambled siRNA or PEX19 siRNA1, and 24 h after transfection, the cells were infected with the WSN (H1N1), AH05 (H5N1), or AH13 (H7N9) virus at an MOI of 0.01. At 24 and 48 h post infection (p.i.)., the virus titers of the culture supernatants were determined by plaque assay on MDCK cells.

### 2.9. Generation of PEX19_KO A549 Cells and Virus Infection

The single guide RNA (sgRNA) sequence (5′-CACCGGTGTCGGGGCCGAAGCGGAC-3′) targeting the PEX19 gene was designed by using an online CRISPR Design tool (Dr. Feng Zhang’s lab website) and inserted into the pSpCas9(BB)-2A-GFP (pX458) vector. The resulting construct was electrotransfected into A549 cells by using the Neon Transfection System (ThermoFisher Scientific, Waltham, MA, USA). At 48 h post transfection, the transfected cells were trypsinized, and the single GFP-positive cells were seeded into each well of a 96-well plate using the MoFlo XDP cell sorter. Each of the formed colonies was propagated in 24-well plates, and the knockout of PEX19 was confirmed by Western blotting. The established PEX19_KO and control A549 cells were infected with the WSN (H1N1) virus (MOI = 0.01) to assess the effect of PEX19 knockout on viral replication. At 24 and 48 h p.i., the virus titers of the culture supernatants were determined by plaque assays on MDCK cells.

### 2.10. Establishment of an A549 Stable Cell Line Overexpressing PEX19 and Virus Infection

A PEX19-overexpressing cell line was constructed using the lentiviral vector system (Clontech). HEK293T cells were co-transfected with pLVX-PEX19-IRES-ZsGreen1 plasmids and helper plasmids pSPAX2 and pMDG for packaging lentivirus, and the insert-free pLVX-IRES-ZsGreen1 was transfected as a control. At 48 h post transfection, viral supernatants were collected and used to transduce A549 cells. Forty-eight hours later, the transduction was repeated to enrich transductants. Then, 48 h after the second transduction, GFP-positive cells were collected using a MoFlo XDP cell sorter (Beckman Coulter, Brea, CA, USA), propagated, and detected for PEX19 overexpression by Western blotting. To investigate the effect of PEX19 overexpression on IAV growth, the WSN (H1N1) virus was used to infect PEX19-overexpressing or control A549 cells at an MOI of 0.01. At 24 and 48 h p.i., supernatants were collected and titrated for viral growth titers by plaque assays on MDCK cells.

### 2.11. Plaque Assay

Plaque assays were performed on MDCK cells in 12-well plates as described previously [20].

### 2.12. Peroxisome and Reactive Oxygen Species (ROS) Detection

Peroxisomes in PEX19 siRNA1- or scrambled siRNA-treated A549 cells, or WSN (H1N1)-infected A549 cells were visualized by immunofluorescence microscopy using antibodies against peroxisomal protein PMP70. The profiling of ROS formation by fluorescence microscopy was achieved in WSN (H1N1)-infected or PEX19 siRNA1-treated A549 cells and HEK293T cells loaded with ROS detection reagents (ab139476; Abcam) at 24 h p.i. or 24 h post transfection.

### 2.13. Confocal Microscopy

A549 cells of approximately 70% confluency grown in glass-bottom dishes were transfected with plasmids expressing Myc-PEX19 and Flag-WSN (H1N1) M2 using Lipofectamine 3000 and plus reagent (Invitrogen) or were infected with the WSN (H1N1) virus at an MOI of 2. Cells were washed with PBS at 24 h post transfection or at the indicated time points post infection, fixed with 4% paraformaldehyde (PFA) for 30 min at room temperature, and permeabilized with 0.5% Triton X-100 for 30 min. After blocking with 5% BSA in PBS for 1 h at RT, the cells were incubated with mouse anti-M2 mAb (1:200) and rabbit anti-PEX19 pAb (1:200) for 4 h at RT, followed by washing with PBS three times. The cells were then incubated with secondary antibodies Alexa Fluor 488 goat anti-mouse IgG (H+L) and Alexa Fluor 633 goat anti-rabbit IgG (H+L) (1:400) for 1 h at RT. After three washes with PBS, the cells were examined by using ZEISS LSM 880 Confocal Laser Scanning Microscopy with Fast Airyscan (Zeiss, Oberkochen, Germany). A 3D analysis of images was performed using the Colocalization Finder of Image J 1.54i.

### 2.14. Quantitative Reverse Transcription PCR (RT-qPCR) Analysis

RT-qPCR was used to determine the relative mRNA levels. Total RNA was extracted with TRIzol reagent (Invitrogen) according to the manufacturer’s instructions. RNA was reverse transcribed with an oligo(dT) primer or a random primer. Real-time PCR was performed in QuantStudio 5 (Applied Biosystems, Carlsbad, CA, USA), and β-actin was used as an internal control. The primers used are available upon request.

### 2.15. Statistical Analysis

The results were analyzed for statistical significance by using the two-tailed unpaired Student’s *t*-test with GraphPad Prism software 8.0 (GraphPad, San Diego, CA, USA). A *p*-value of <0.05 was considered significant.

## 3. Results

### 3.1. Identification of PEX19 as a Potential Interacting Host Factor of IAV M2 by the Yeast Two-Hybrid System

We used the yeast two-hybrid system to identify host cellular proteins that interact with the IAV M2 protein. The full-length M2 of A/Sichuan/1/2009 (SC09, H1N1) was used as bait. After selection on SD/–4/X/A (–Ade/–His/–Leu/–Trp/X-a-Gal/AbA) plates, positive clones were obtained. Plasmids were isolated and sequenced after the putative positive clones were grown in an SD/–2 (–Leu/–Trp) medium to identify the potential M2 interactants.

**Figure 1:**
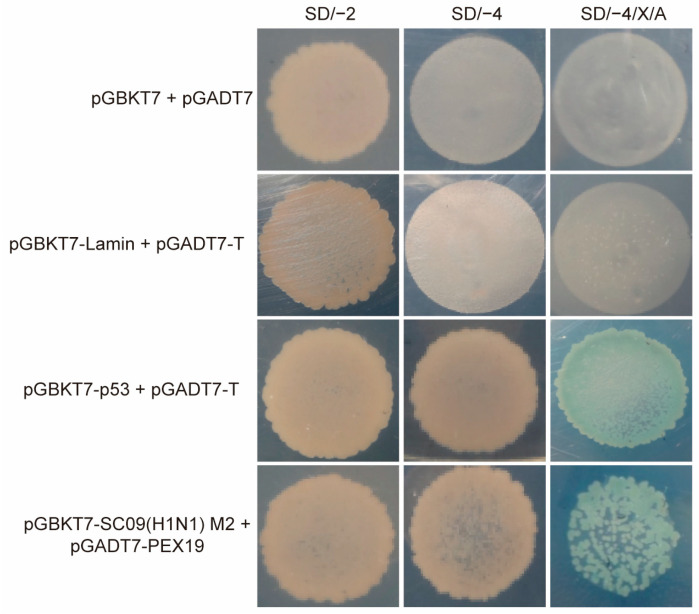
The yeast two-hybrid screen identifies PEX19 as an interacting partner of IAV M2. The yeast strain Y2H Gold was co-transformed with the bait plasmid pGBKT7-SC09(H1N1) M2, containing SC09(H1N1) M2 fused to the GAL4-binding domain (BD) in a pGBKT7 vector, together with the prey plasmid pGADT7-PEX19, which encodes PEX19 fused to the Gal4-activation domain (AD). Positive protein–protein interactions are indicated by the growth of blue colonies in the SD/–4/X/A plates in the presence of X-a-Gal. The co-transformation of pGBKT7-p53 encoding the Gal4-BD fused with murine p53 and pGADT7-T encoding the Gal4-AD fused with SV40 large T-antigen served as a positive control. The co-transformation of pGBKT7-Lamin, which encodes the Gal4-BD fused with Lamin and pGADT7-T, served as a negative control. SD/–2: SD/–Leu/–Trp; SD/–4: SD/–Ade/–His/–Leu/–Trp; and SD/–4/X/A: SD/–Ade/–His/–Leu/–Trp/X-a-Gal/AbA.

One specific clone from this screen was discovered to bear the full-length ORF of PEX19. Its role in the modulation of IAV replication has never been documented. PEX19-M2 interaction was then retested by co-transforming yeast with the pGBKT7-SC09(H1N1) M2 bait plasmid and pGADT7-PEX19 prey plasmid. As shown in Figure 1, PEX19 specifically interacted with M2 in yeast.

### 3.2. IAV M2 Interacts with PEX19 in Human Cells

**Figure 2:**
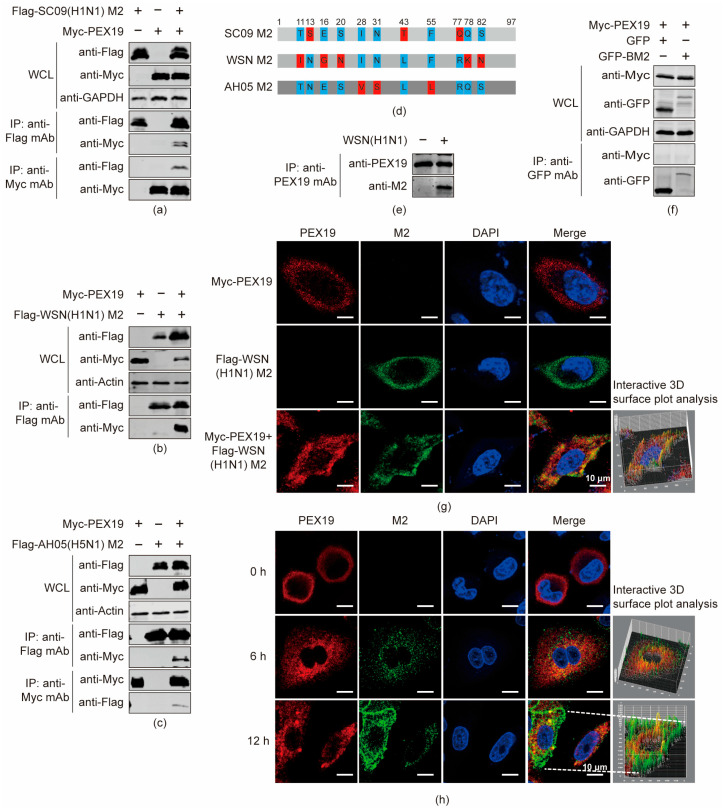
IAV M2 protein interacts with host factor PEX19. (**a**–**c**) HEK293T cells were co-transfected with the indicated combination of plasmids expressing Myc-tagged PEX19 and Flag-tagged SC09 (H1N1) M2 (**a**), WSN (H1N1) M2 (**b**), or AH05 (H5N1) M2 (**c**). Cell lysates were subjected to immunoprecipitation with mouse anti-Flag or anti-Myc mAb, and the bound proteins were detected by Western blotting. (**d**) Schematic representation of differences in amino acid sequences of M2. (**e**) A549 cells were infected with the WSN (H1N1) virus at an MOI of 2 for 24 h. Cell lysates were immunoprecipitated with a mouse anti-PEX19 mAb, and the bound proteins were detected by Western blotting. (**f**) HEK293T cells were co-transfected with plasmids expressing Myc-PEX19 and GFP or GFP-tagged BM2. Cell lysates were immunoprecipitated with a mouse anti-GFP mAb, and the bound proteins were detected by Western blotting. (**g**,**h**) A549 cells were transfected individually or in combination with plasmids expressing Flag-tagged WSN (H1N1) M2 and Myc-tagged PEX19 (**g**) or were infected with the WSN (H1N1) virus at an MOI of 1 (**h**). Cells were immunostained with anti-M2 and anti-PEX19 antibodies 24 h after transfection or the indicated timepoints p.i., and were visualized by confocal microscopy. The 3D analysis of images was performed using Image J.

To examine whether M2 interacts with PEX19 in human cells, we performed co-IP experiments in HEK293T cells that were transfected individually or in combination with plasmids expressing the Flag-tagged SC09 (H1N1) M2 protein and Myc-tagged PEX19 protein. Cell lysates were subjected to immunoprecipitation (IP) with a mouse anti-Flag mAb or a mouse anti-Myc mAb, followed by Western blotting with a rabbit anti-Flag pAb and a rabbit anti-Myc pAb. As shown in Figure 2a, the M2 of the SC09 (H1N1) virus interacted with PEX19 in HEK293T cells. The physical interaction between PEX19 and M2 in transiently transfected HEK293T cells was also confirmed with the M2 of the A/WSN/33 (WSN, H1N1) (Figure 2b) and AH05 (H5N1) viruses (Figure 2c) that bear eight or six substitutions compared to the M2 of the SC09 (H1N1) virus (Figure 2d), indicating that the interaction between PEX19 and M2 is a property shared by distinct IAV strains.

We also performed a co-IP experiment in A549 cells that were mock-infected or infected with the WSN (H1N1) virus at an MOI of 2. At 24 h p.i., cell lysates were immunoprecipitated with a mouse anti-PEX19 mAb, followed by Western blotting with a rabbit anti-PEX19 pAb and a mouse anti-M2 mAb. The results showed that the viral M2 protein interacted with endogenous PEX19 in WSN (H1N1)-infected cells (Figure 2e).

To further determine whether the interaction between PEX19 and M2 can be extended to the influenza B virus (IBV), a co-IP experiment was performed to examine whether PEX19 interacts with the BM2 of the B/Jilin/20/2003 virus. As shown in Figure 2f, PEX19 was not coimmunoprecipitated with a green fluorescent protein (GFP)-tagged BM2. Notably, the BM2 of the B/Jilin/20/2003 virus contains 109 amino acid residues and shows only 12.4% homology with SC09 (H1N1) M2, although the IAV M2 and BM2 are thought to play common roles in the life cycle of the IAV and IBV. This result indicates the interaction specificity of PEX19 with the M2 protein of the IAV.

Immunofluorescence and confocal microscopy were used to examine the localization of M2 and PEX19 in A549 cells that were transfected individually or in combination with plasmids expressing Flag-tagged WSN (H1N1) M2 and Myc-tagged PEX19 (Figure 2g) or were infected with the WSN (H1N1) virus at an MOI of 1 (Figure 2h). At 24 h post transfection, we observed clear colocalization between WSN (H1N1) M2 and PEX19 in transiently transfected A549 cells. M2 and PEX19 were also partially colocalized at 6 and 12 h p.i. in WSN (H1N1) virus-infected A549 cells.

### 3.3. The Cytoplasmic Tail Domain of M2 and the C Terminus of PEX19 Are Key Regions of Interactions

**Figure 3:**
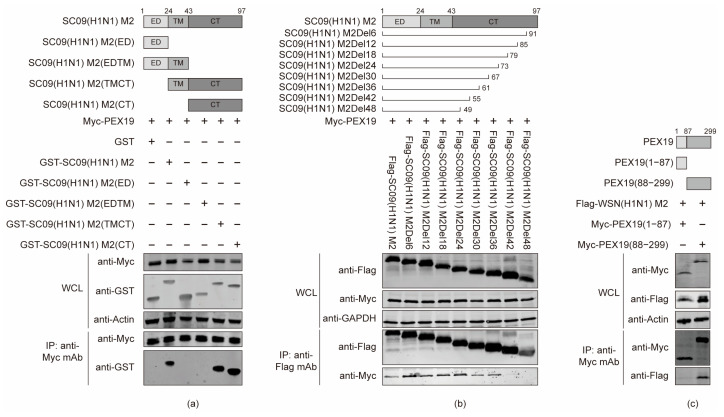
The cytoplasmic tail domain of M2 and the C terminus of PEX19 are key regions of interactions. (**a**–**c**) HEK293T cells were co-transfected with the indicated combinations of plasmids. At 48 h post transfection, cell lysates were immunoprecipitated with a mouse anti-Myc mAb (**a**,**c**), or a mouse anti-Flag mAb (**b**), and the bound proteins were detected by Western blotting with a rabbit anti-GST pAb and a rabbit anti-Myc pAb (**a**) or with a rabbit anti-Flag pAb and a rabbit anti-Myc pAb (**b**,**c**).

To define the specific structural domain and residue of IAV M2 that mediate the binding with PEX19, SC09 (H1N1) M2 was truncated into four fragments according to its function, i.e., ED, ED-TM, TM-CT, and CT. Full-length M2 and the four fragments were fused to an N-terminal GST tag for the generation of protein expression constructs. We then performed co-IP experiments in HEK293T cells that were co-transfected with these fusion constructs and Myc-PEX19-expressing plasmids. We found that the full-length M2, M2 CT, and M2 TM-CT of the SC09 (H1N1) virus interacted with PEX19, but the ED and ED-TM fragments of M2 were not able to associate with PEX19 (Figure 3a), indicating that the M2 CT domain is responsible for the interaction with PEX19.

Next, we endeavored to narrow down the M2 CT region that is involved in the interaction with PEX19. M2 truncation mutants with 6-, 12-, 18-, 24-, 30-, 36-, 42-, and 48-amino-acid deletions from the C terminus were generated and used in co-IP assays. As shown in Figure 3b, PEX19 was co-immunoprecipitated with full-length SC09 (H1N1) M2 and all truncation mutants, except M2Del42 and M2Del48, indicating that the amino acids at positions 56–61 of M2 are important for the interaction with PEX19.

Similarly, PEX19 was divided into two structural fragments, i.e., PEX19 (1–87) and PEX19 (88–299), to examine their interaction with M2 by co-IP experiments. As shown in Figure 3c, PEX19 (88–299), but not PEX19 (1–87), was responsible for the interaction with M2.

### 3.4. PEX19 Inhibits the Replication of IAV

**Figure 4:**
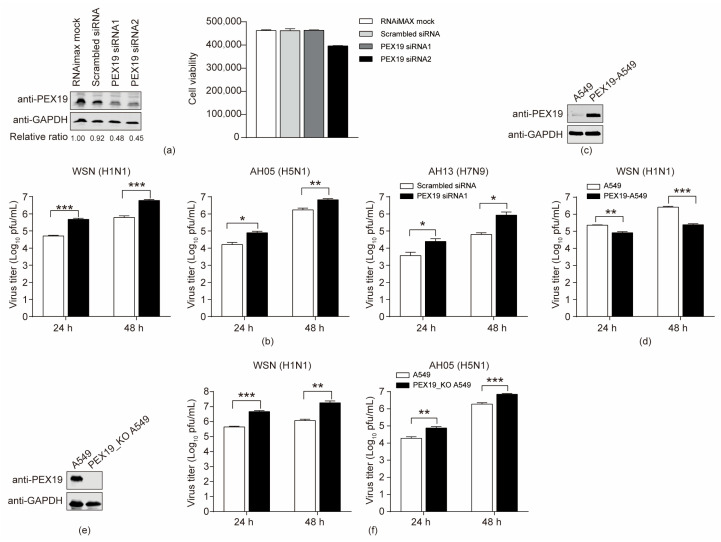
PEX19 inhibits the replication of IAV. (**a**) A549 cells were transfected with siRNA targeting PEX19 or scrambled siRNA for 48 h. Whole-cell lysates were then analyzed by Western blotting with a mouse anti-PEX19 mAb. The band intensities of PEX19, quantified using ImageJ, were normalized to GAPDH and are expressed as relative ratios compared to RNAimax-treated cells. The cell viability of siRNA-treated A549 cells was measured using the CellTiter-Glo assay. The data are presented as means ± standard deviations for triplicate transfections. (**b**) PEX19 siRNA1- or scrambled siRNA-transfected A549 cells as in (**a**) were infected with the WSN/33 (H1N1), AH05 (H5N1), or AH13 (H7N9) virus. At 24 and 48 h p.i., supernatants were titrated for infectious viruses by plaque assays on MDCK cells. (**c**) The stable overexpression of PEX19 in PEX19-overexpressing cells was confirmed by Western blotting with a rabbit anti-PEX19 pAb. (**d**) The WSN (H1N1) virus was used to infect the PEX19-overexpressing or control A549 cells at an MOI of 0.01. Supernatants were collected at 24 and 48 h p.i., and virus titers were determined by plaque assays on MDCK cells. (**e**) A stable PEX19_KO A549 cell line was established by the CRISPR/Cas9 system, and the knockout of PEX19 was confirmed by Western blotting with a rabbit anti-PEX19 pAb. (**f**) PEX19_KO or control A549 cells were infected with the WSN/33 (H1N1) or AH05 (H5N1) virus. At 24 and 48 h p.i., supernatants were titrated for infectious viruses by plaque assays on MDCK cells. Three independent experiments were performed in (**b**,**d**,**f**), and data are shown as means ± standard deviations for triplicates from a representative experiment. *, *p* < 0.05; **, *p* < 0.01; and ***, *p* < 0.001.

Given that both endogenous and exogenous PEX19 interacted with IAV M2, we then determined to investigate the role of PEX19 in the replication of IAV in A549 cells. A549 cells were treated with two specific siRNAs targeting PEX19 or scrambled siRNA for 48 h. Western blotting showed that the treatment with both PEX19 siRNA1 and siRNA2 significantly reduced the expression level of PEX19 compared to scrambled siRNA-treated cells, and PEX19 knockdown by siRNA1 produced no visible adverse effect on cell viability (Figure 4a). The scrambled siRNA or PEX19 siRNA1-treated cells were infected with the WSN (H1N1), AH05 (H5N1), or AH13 (H7N9) virus at an MOI of 0.01, and the culture supernatants were subject to plaque assays at 24 and 48 h p.i. for virus titration. Compared to scrambled siRNA-treated cells, PEX19 silencing led to 9.5-/9.8-, 4.9-/4.0-, and 6.7-/13.7-fold increases in the growth titers of the WSN (H1N1), AH05 (H5N1), and AH13 (H7N9) viruses at 24 and 48 h p.i., respectively (Figure 4b). These results indicate that PEX19 downregulation promotes IAV replication.

Subsequently, we investigated the impact of PEX19 overexpression on IAV replication. To this end, we established a lentiviral vector-based A549 cell line that overexpresses PEX19. Western blotting showed that PEX19 was dramatically overexpressed in PEX19-overexpressing cells compared to empty retrovirus-transduced control cells (Figure 4c). The PEX19-overexpressing and control A549 cells were infected with the WSN (H1N1) virus at an MOI of 0.01, and the supernatants were titrated for infectious viruses at 24 and 48 h p.i. As shown in Figure 4d, the overexpression of PEX19 resulted in 2.7- and 10.8-fold reductions in virus growth titers.

To further validate the role of PEX19 in restricting the replication of the IAV, we also generated a PEX19 knockout (PEX19_KO) A549 cell line by using the CRISPR/Cas9 system. Western blotting showed that PEX19 was completely knocked out in PEX19_KO cells (Figure 4e). The PEX19_KO and control A549 cells were infected with the WSN (H1N1) or AH05 (H5N1) virus (MOI = 0.01), and the supernatants were subject to plaque assays at 24 and 48 h p.i. We found that PEX19 knockout enhanced the growth titers of the WSN (H1N1) and AH05 (H5N1) viruses by 10.5-/15.3- and 4.0-/3.7-fold at 24 and 48 h p.i., respectively (Figure 4f).

Together, these results indicate that PEX19 is a negative regulator of IAV replication.

### 3.5. IAV Infection Decreases the Pool of Peroxisomes in A549 Cells

**Figure 5:**
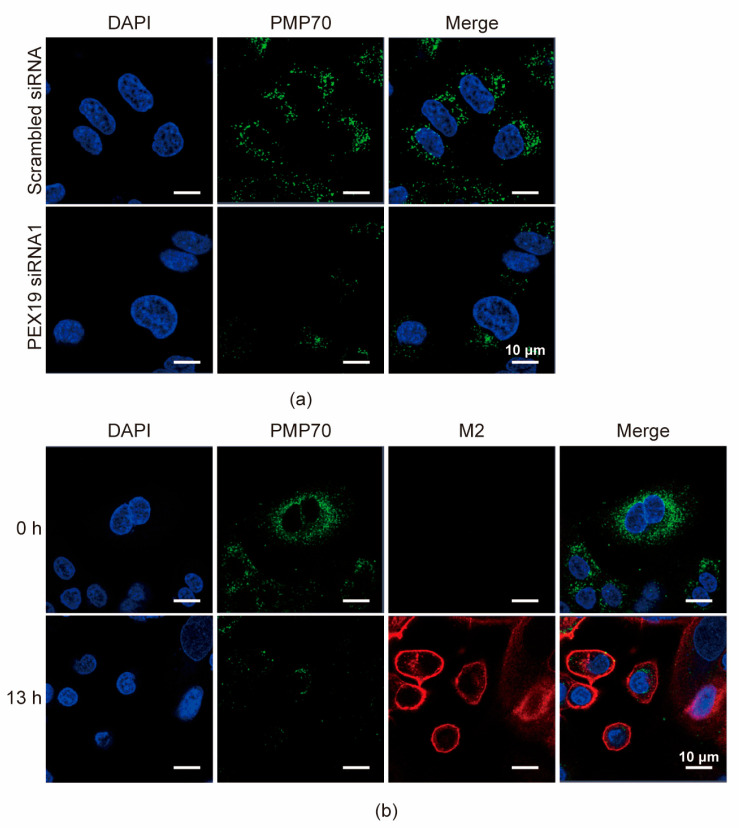
PEX19 knockdown and IAV infection decrease the pool of peroxisomes in A549 cells. (**a**) A549 cells were transfected with PEX19 siRNA1 or scrambled siRNA for 24 h and were then subjected to immunofluorescence microscopy analysis using a mouse anti-PMP70 mAb. (**b**) A549 cells were infected with the WSN (H1N1) virus (MOI = 1) and were then subjected to immunofluorescence microscopy analysis using a mouse anti-PMP70 mAb and a rabbit anti-M2 pAb at 0 and 13 h p.i.

Due to the critical role of PEX19 in peroxisome biogenesis, we first examined the effect of PEX19 knockdown on the number of peroxisomes. A549 cells were transfected with PEX19 siRNA1 or scrambled siRNA for 24 h, and the numbers of peroxisomes were then visualized by immunofluorescence assays with antibodies against the peroxisome membrane protein PMP70. We found that the numbers of peroxisomes were significantly reduced in PEX19 siRNA1-treated cells compared to scrambled siRNA-treated cells (Figure 5a), indicating that PEX19 knockdown downregulates the pool of peroxisomes. Next, we also determined whether IAV infection causes any change in the number of peroxisomes. A549 cells were infected with the WSN (H1N1) virus at an MOI of 1. At 0 and 13 h p.i., the cells were visualized by confocal microscopy with antibodies against PMP70 and M2. We found that the number of peroxisomes was significantly decreased in A549 cells at 13 h p.i. compared to 0 h p.i. (Figure 5b), demonstrating that IAV infection reduces the abundance of peroxisomes.

### 3.6. PEX19 Deficiency Promotes Cell Damage in IAV-Infected Cells by Inducing ROS and Compromising the ROS-Processing Function of Peroxisomes

**Figure 6:**
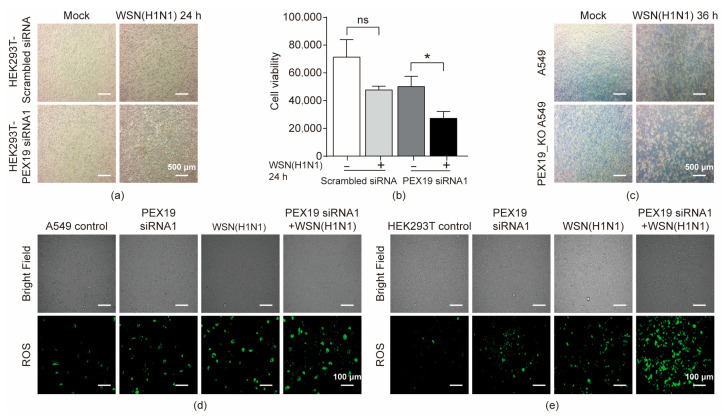
PEX19 deficiency promotes cell damage in IAV-infected cells by inducing ROS and compromising the ROS-processing function of peroxisomes. (**a**) HEK293T cells were treated with PEX19 siRNA1 or scrambled siRNA for 12 h, followed by mock infection or infection with the WSN (H1N1) virus (MOI = 1). Bright-field images were acquired under an inverted microscope at 24 h p.i. (**b**) The viability of HEK293T cells treated as in (**a**) was analyzed by a CellTiter-Glo assay at 24 h p.i. *, *p* < 0.05 and ns, not significant. (**c**) PEX19_KO and control A549 cells were mock-infected or infected with the WSN (H1N1) virus (MOI = 1). Bright-field images were acquired under an inverted microscope at 36 h p.i. (**d**,**e**) A549 (**d**) and HEK293T (**e**) cells were left untreated or were treated with PEX19 siRNA1 for 12 h, followed by infection with the WSN (H1N1) virus (MOI = 1). The profiling of ROS formation was visualized by fluorescence microscopy with ROS detection reagents at 24 h p.i.

IAV infection activates the production of reactive oxygen species (ROS), which contributes to the replication and pathogenicity of the IAV [32]. Peroxisome, containing oxidoreductases for scavenging ROS, is essential for balancing intracellular ROS levels [33,34]. Given that IAV infection led to a decrease in peroxisome abundance, we then attempted to investigate whether IAV infection induces cell damage by affecting PEX19. HEK293T cells were treated with PEX19 siRNA1 or scrambled siRNA for 24 h, followed by infection with the WSN (H1N1) virus at an MOI of 1. We found that cell damage, observed using an optical microscope, was more significantly pronounced in PEX19 siRNA1-treated cells than that in the scrambled siRNA-treated cells at 24 h p.i. (Figure 6a). In addition, cell viability was more severely compromised in PEX19 siRNA1-treated cells compared to the scrambled siRNA-treated cells at 24 h p.i. (Figure 6b). Consistently, IAV infection also led to more severe cell damage in PEX19_KO A549 cells than in control cells (Figure 6c).

Next, we further defined the relationship between PEX19 expression and ROS production during IAV infection. A549 cells (Figure 6d) and HEK293T cells (Figure 6e) were left untreated or were treated with the PEX19 siRNA1 for 12 h, followed by infection with the WSN (H1N1) virus (MOI = 1). The profiling of ROS formation by fluorescence microscopy was performed at 24 h p.i. with ROS detection reagents. We found that IAV infection or PEX19 knockdown increased the cellular level of ROS and that IAV infection and PEX19 knockdown synergistically resulted in a higher ROS level.

Taken together, our results demonstrate that PEX19 downregulation in IAV-infected cells contributes to the induction of cell damage by inducing ROS production.

### 3.7. IAV Infection and PEX19 Knockout Reduce the Levels of Type III Interferons

**Figure 7:**
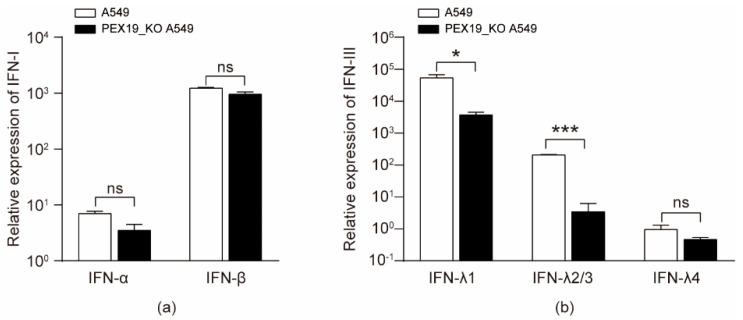
IAV infection and PEX19 knockout reduce the levels of type III interferons. (**a**,**b**) PEX19_KO and control A549 cells were infected with the WSN (H1N1) virus (MOI = 2) for 24 h. The mRNA levels of IFN-α and IFN-β (**a**) or type III IFNs (**b**) were determined by RT-qPCR (*n* = 3). *, *p* < 0.05; ***, *p* < 0.001; and ns, not significant.

IAV infection leads to the activation of innate immunity, producing different types of antiviral interferons [35]. Previous studies have shown that peroxisome is a primary site for the initiation of type III interferon expression for combating invading pathogens [36,37]. Given that IAV infection decreased the pool of peroxisome, we then assessed the effect of IAV infection on the production of type III interferon in PEX19_KO and control A549 cells infected with the WSN (H1N1) virus (MOI = 2). RT-qPCR analyses with the cell lysates showed that the mRNA levels of IFN-α and IFN-β in PEX19_KO A549 cells were unaffected compared to A549 control cells at 24 h p.i., as shown in Figure 7a. We then examined the effect of PEX19 on the production of type III IFNs with the same approach. The results showed that PEX19 knockout was able to reduce the expression of type III IFNs (Figure 7b). These results indicate that the PEX19-dependent intactness of peroxisome is important for type III IFN-mediated innate immunity.

### 3.8. The M2 Protein Disturbs the Interactions between PEX19 and Peroxisome-Associated Factors

**Figure 8:**
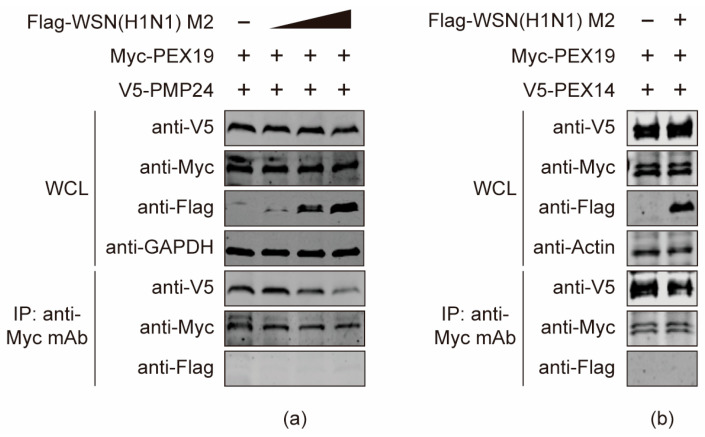
The M2 protein disturbs the interactions between PEX19 and peroxisome-associated factors. (**a**,**b**) HEK293T cells were co-transfected with plasmids expressing Myc-PEX19, V5-PMP24 (**a**), or V5-PEX14 (**b**) and gradually increased amount of Flag-tagged WSN (H1N1) M2. At 24 h post transfection, cell lysates were immunoprecipitated with a mouse anti-Myc mAb, and the bound proteins were Western blotted with a rabbit anti-Flag, anti-Myc, or anti-V5 pAb.

PEX19 is an important molecular chaperone for peroxisome-associated factors. It recognizes and binds PEXs and peroxisome membrane proteins (PMPs) to import them into peroxisomes, thus playing pivotal roles in peroxisome biogenesis. Given that the PEX19-dependent intactness of peroxisome is important for the ROS regulation and type III IFN-mediated innate immunity, we then attempted to investigate whether the binding of M2 to PEX19 would affect the molecular chaperone function of PEX19 on PEXs and PMPs. HEK293T cells were co-transfected with plasmids expressing Myc-PEX19, V5-PMP24 and gradually increased amount of Flag-tagged WSN (H1N1) M2. At 24 h post transfection, cell lysates were subjected to co-IP with a mouse anti-Myc mAb, and the bound proteins were detected by Western blotting. As shown in Figure 8a, the presence of M2 interfered with the interaction between PEX19 and PMP24. Notably, the interaction between PEX19 and M2 was also dramatically affected by PMP24, demonstrating that there is a mutual interference between M2 and PMP24 in the interaction with PEX19. Subsequently, we performed co-IP experiments to examine the effect of M2 on PEX19/PEX14 binding. We found that the association between PEX19 and PEX14 was also affected by the presence of M2 (Figure 8b). Taken together, these results indicate that M2 disrupts the process by which PEX19 binds and transports peroxisome-associated proteins, which may have an impact on the biogenesis and function of peroxisomes.

## 4. Discussion

The limitation of viral proteins encoded by the genome of the IAV implies that the viral components must interact extensively with the host cellular machinery to achieve efficient viral replication. Acting in several different stages of the IAV replication cycle, the viral M2 protein interacts with a couple of host cellular proteins, e.g., LC3 [15], Atg6/Beclin-1 [16], UBR4 [19], and TRAPPC6Adelta [20], to facilitate the replication of the IAV. The identification of novel host factors interacting with M2 and the elucidation of underlying mechanisms would expand the function landscape of M2 in the course of IAV infection.

In the present study, we investigated the interaction between IAV M2 and PEX19, a host factor identified from a Y2H system screen. We found that both endogenous and exogenous PEX19 interacted with M2. A detailed analysis showed that the CT region of M2 is responsible for mediating the interaction with the C terminus of PEX19. The C terminal globular domain of PEX19 is responsible for identifying and binding PMPs [38]. It contains a farnesylation box (CAAX) that functions to farnesylate PEX19, thereby facilitating its functions of helping PMPs insert into the phospholipid bilayer of the peroxisome [39].

By modulating the expression level of PEX19, we found that the siRNA knockdown or CRISPR/Cas9 knockout of PEX19 expression enhanced the growth titers of the IAV in cell culture, whereas the overexpression of PEX19 led to reduced progeny virus yield. These results indicated that PEX19 functions to restrict the replication of the IAV. We subsequently explored how IAV infection affects the function of PEX19.

As our confocal microscopy analysis revealed that PEX19 and M2 were colocalized in the cytoplasm, we speculated that the interaction between M2 and the C terminus of PEX19 in the cytoplasm may interfere with the recognition and binding of PEX19 with PEXs and PMPs, thereby disrupting peroxisome biosynthesis. This hypothesis was validated by investigating the interaction between PEX19 and PMP24 or PEX14.

PMP24 is an important component of the peroxisomal membrane and plays a role in liver lipid metabolism [36]. We found that the co-expression of M2 interfered with the interaction between PEX19 and PMP24, and the M2-PEX19 interaction was also antagonized by PMP24. This finding implies that IAV M2 may compete with PMP24 for the binding with PEX19, which may affect the assembly of peroxisomes.

PEX14 is indispensable for the import of matrix proteins during peroxisome biosynthesis [40]. It is introduced into the peroxisomal membrane by PEX19 and is subsequently docked with the matrix protein receptor PEX5 to complete the import of peroxisomal matrix proteins [41]. It has been reported that the loss of PEX19 causes the aggregation of PEX14 on lipid droplets, thereby failing to be inserted into the peroxisomal membrane [42]. In the present study, we found that IAV M2 weakened the binding of PEX19 with PEX14. This interfering role of M2 may also impair the insertion of PEX14 into the peroxisomal membrane, thus affecting the import of peroxisomal matrix proteins. The specific mechanism remains to be further explored.

Consistent with the importance of PEX19 in the biogenesis of peroxisomes, we found that the number of peroxisomes was significantly decreased in IAV-infected A549 cells. Given the importance of peroxisomes in controlling the metabolism of ROS, a reduction in peroxisomes in the course of IAV infection led to enhanced levels of ROS and subsequent cell damage.

It has been shown that peroxisomes are important platforms for mediating early innate immunity and are increasingly important in the antiviral immune response [36,37]. Upon virus infection, viral RNA recognizes and binds receptors such as RIG-I and MAD5 and then binds to MAVS in peroxisomes (peroxisome MAVS), leading to the IRF1-dependent production of type III IFNs to counteract viral infection [43]. We found that the transcription of IFN-α and IFN-β was unaffected upon IAV infection in PEX19_KO versus control A549 cells. By contrast, PEX19 knockout led to a reduction in type III IFNs in IAV-infected cells. These results demonstrate the importance of PEX19 in peroxisome MAVS-mediated type III IFN response during IAV infection.

## 5. Conclusions

**Figure 9:**
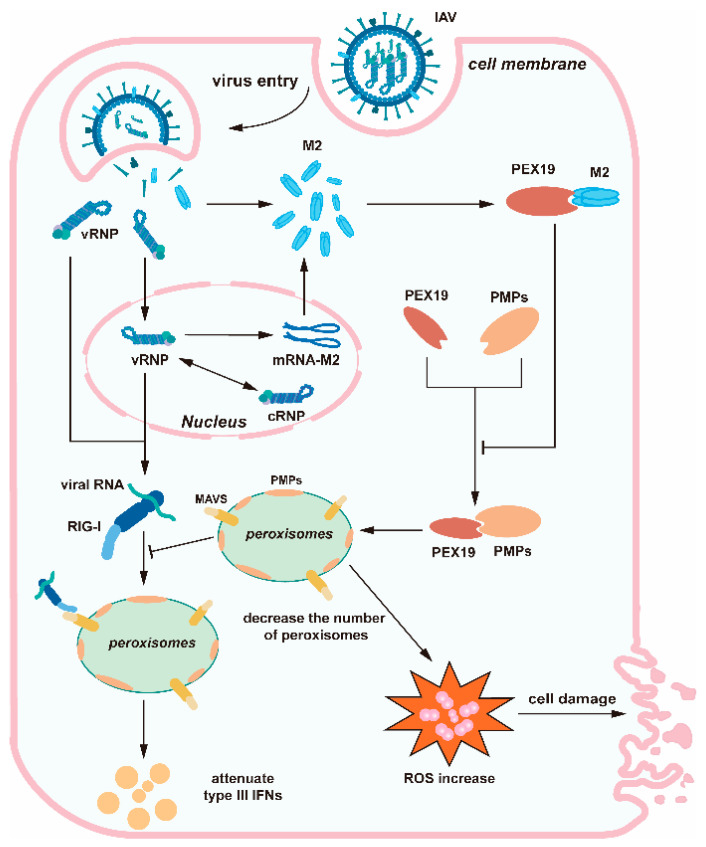
Schematic diagram showing the role of PEX19 during IAV replication. IAV M2 interferes with the interactions between PEX19 and peroxisomal proteins, which may lead to a decrease in the peroxisome pool during IAV infection. The decrease in peroxisomes during IAV infection causes the accumulation of reactive oxygen species (ROS) and cell damage. Meanwhile, the IAV-induced reduction in peroxisome quantity also compromises the peroxisome MAVS-mediated type III IFN responses, which provides an environment that favors viral propagation.

In summary, we identified a novel host factor, i.e., PEX19, that interacts with IAV M2 and revealed the involvement of PEX19 in the negative regulation of IAV replication. IAV M2 was able to inhibit the interactions of PEX19 with peroxisomal proteins, which may reduce the abundance of peroxisomes. The decrease in the peroxisome pool during IAV infection induced intracellular ROS accumulation and caused cell damage. In addition, the IAV-induced reduction in the number of peroxisomes also suppressed peroxisome MAVS-mediated type III IFN responses, thus creating a favorable condition for viral replication (Figure 9).

## Data Availability

The data presented in the study are included in the article, further inquiries can be directed to the corresponding authors.
